# A Giant Occipital Encephalocele

**Published:** 2010-12-01

**Authors:** Amit Agarwal, Aruna Vijay Chandak, Anand Kakani, Shivshankar Reddy

**Affiliations:** Department of Neurosurgery, Datta Meghe Institute of Medical Sciences Sawangi, Wardha, India

**Keywords:** Microcephaly, Occipital encephalocele, Neurological development

## Abstract

Giant occipital encephaloceles are rare lesions. Because of their enormous size they pose a surgical challenge. Herein we report a four months old female baby who presented with progressively increasing swelling over the occipital region. This swelling was present since birth. Surgery was planned to reduce the size of the swelling as well as its contents. The redundant sac was excised and reduced sufficiently enough to accommodate the healthy looking brain tissue. In contrast to the previous case reports where the neonates had poor prognosis, this infant did well postoperatively.

## INTRODUCTION

Occipital encephaloceles can vary from a small pedunculated swelling with a narrow neck to an extremely large swelling. In one study up to 16% of the occipital encephaloceles were more than 20 cm in diameter [1].

In giant occipital encephaloceles the size of the swelling is larger than the size of the head from which they arise, and because of their enormous size these poses a surgical challenge [2-7]. We report this case to highlight the difficulties in the management of giant occipital encephaloceles. 

## CASE REPORT

A four months female baby presented with progressively increasing swelling over the occipital region since birth. The baby was born at 37 weeks of gestation by normal delivery with a birth weight of 2400 gms.

There was no abnormality on physical examination except for a large cystic mass in the occipital region. It was larger than the size of the head (Fig. 1). The skin over the swelling was stretched but well formed. The anterior fontanelle was closed. Baby was able to track objects and light and pupils were reactive. Routine hematological and biochemical investigations were reported as normal. A computerized tomography scan (CT) demonstrated the encephalocele with evidence of herniation of very thin looking redundant brain tissue into the sac (Fig. 2,3). CT images also revealed a significant defect of the occipital bone with well formed parietal bones.

**Figure:**
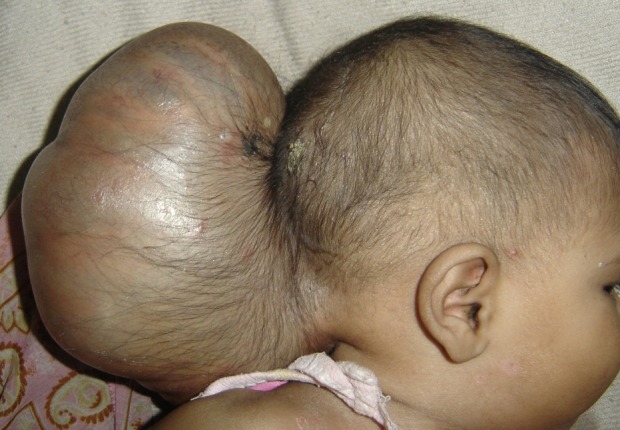
Figure 1: Clinical photograph showing giant occipital encephalocele.

**Figure:**
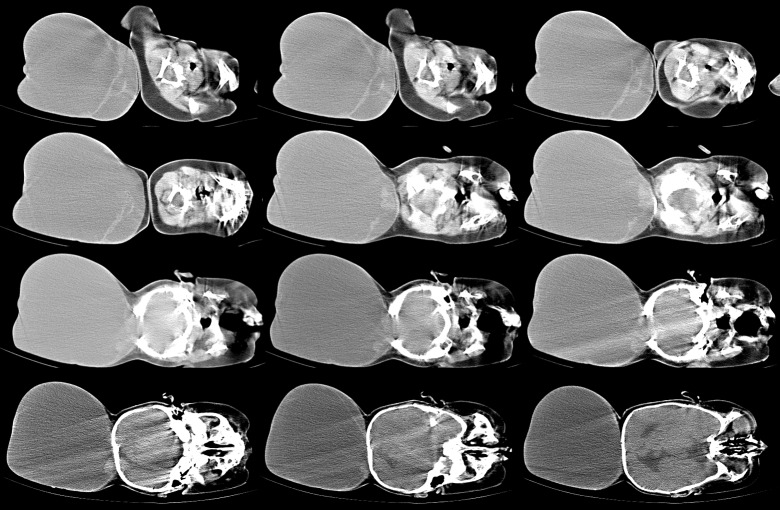
Figure 2: CT scan brain showing large encephalocele sac with protrusion of the contents.

**Figure:**
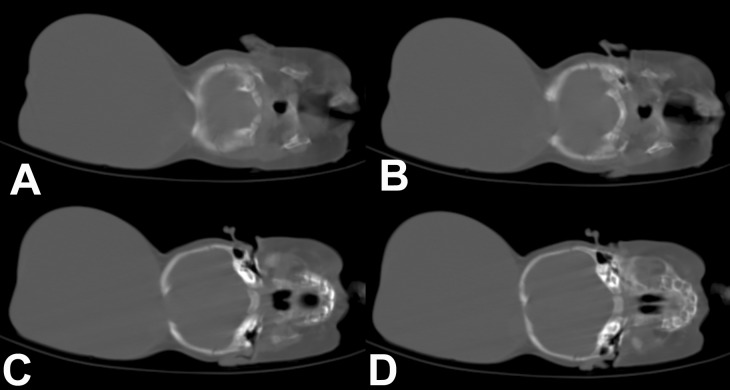
Figure 3: CT scan brain (bone window) showing large defect in the occipital bone with sclerosed margins.

Surgery was planned to reduce the size of the swelling as well as its contents. At operation patient was positioned in lateral position. A circumferential incision was placed over the sac, and the neck was dissected out. Sac was then opened. The herniated brain tissue looked redundant. However, near to opening in the skull there was evidence of normal looking arterioles and veins on the surface of the occipital lobe with normal sulci and gyri pattern. The sac was reduced in size, sufficient enough to accommodate the healthy looking brain tissue. The skin was closed with interrupted sutures (Fig. 4). There was evidence of persistent cystic collection in the occipital region with mild dilatation of ventricles. Child remained well in follow up and monitored for fluid collection and the requirement for a shunt.

**Figure:**
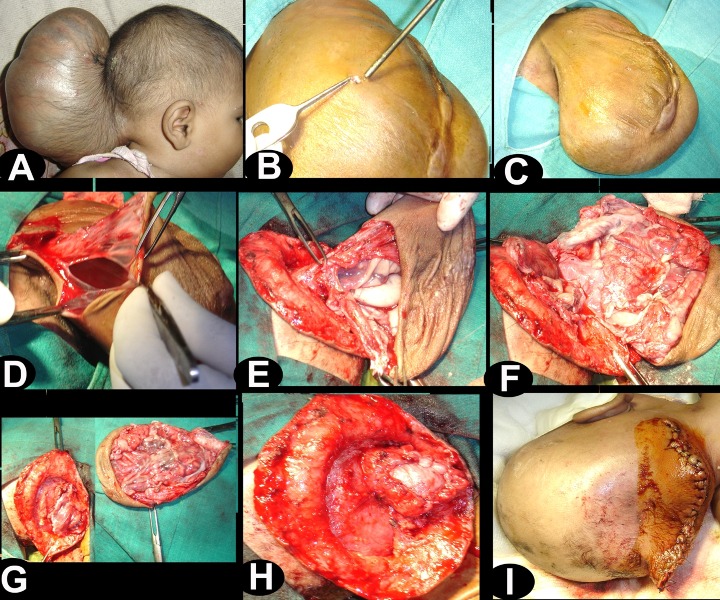
Figure 4: Intra-operative photographs showing operative steps (B) small amount of the CSF was let out through a small opening in the sac, (C) note the reduction in the size of swelling, (D) sac was opened, (E) base of sac was defined, note the presence of large vessels near to the opening in the skull, (F and G) redundant brain tissue was excised while preserving the large vessels, (H and I) dura and skin were closed.

## DISCUSSION

Encephaloceles account for 10 to 20% of all craniospinal dysraphisms [8] and 70% of occipital encephaloceles occur in females [1]. These lesions are usually covered either with normal skin, dysplastic skin or a thin, distorted meningeal membrane. The large sized swellings may have significant brain herniation, abnormality of the underlying brain, microcephaly and ventriculomegaly. Such patients usually have poor prognosis [1]. Encephaloceles with a small amount of dysfunctional tissue are conventionally treated by excision of the herniated brain tissue and repair of the dural defect. The surgical management of children with large defect along with herniation of a considerable proportion of brain matter into the sac, at times can be extremely difficult. In such cases preservation of the herniated brain parenchyma can be accompanied by expansile cranioplasty [6-10].

Patients with giant encephalocele and large amount of brain tissue in the sac usually die either shortly after birth or as a result of operation. A microcephalic child with neurological de?cit and a sac containing cerebrum, cerebellum and brain stem structures, carry a poor prognosis [2,6]. In such patients, it is generally impossible to foretell whether the infant will die quickly or will continue to live for many months or years, as size of the encephalocele itself is not a guide to prognosis. Ultimate result depends on the amount of normal brain tissue left inside the skull after the operation. Surgery thus just facilitates nursing of the baby [6].

In contrast to the previous case reports where the neonate had poorer prognosis (because of larger lesions and significant brain tissue within the sac) this infant was neurologically well developed [2]. Furthermore less functional tissue in the sac made the surgical excision of the sac easy and safe.

## Footnotes

**Source of Support:** Nil

**Conflict of Interest:** None declared
